# Risk assessment of malaria importation to Europe and other non-endemic regions via air-travel

**DOI:** 10.1186/1475-2875-13-S1-P65

**Published:** 2014-09-22

**Authors:** Dariya Ordanovich, Andrew Tatem

**Affiliations:** 1 University of Southampton, Southampton, UK

## 

Increasing human mobility and the expansion of global air travel has resulted in increasing movements of vector-borne diseases. Despite having eliminated malaria and being classed as ‘malaria-free’, European, North-American and some Asian countries still see tens of thousands of cases each year through importation. This work focuses on the assembly of a global database of nationally reported statistics on imported malaria cases over the past 10 years. We highlight the substantial spatial, temporal and demographic heterogeneities that exist between countries and explore the variations in origins of cases, risk groups and malaria types. Further, we examine the possibility of using a range of widely reported country statistics on socioeconomic factors, travel data and geospatial data on malaria risk to predict the number of cases each country is likely to see through construction of a multivariate statistical model. Results show strong predictive power in determining differences and driving factors between countries in rates of imported malaria. Such a model has the potential to be used for guiding interventions to reduce rates of malaria importation, as well as for future scenario analyses for strategic planning.

